# Four infants presenting with severe vomiting in solid food protein-induced enterocolitis syndrome: a case series

**DOI:** 10.1186/1752-1947-6-160

**Published:** 2012-06-26

**Authors:** Amolak S Bansal, Sree Bhaskaran, Rhea A Bansal

**Affiliations:** 1 Department of Immunology, St Helier Hospital, Wrythe Lane, Carshalton, Surrey, SM5 1AA, UK; 2 Consultant in Immunology and Allergy, St Helier Hospital, Wrythe Lane, Carshalton, Surrey, SM5 1AA, UK

## Abstract

**Introduction:**

Several different foods have been implicated in inducing the delayed and very significant vomiting and sometimes diarrhea that occurs in food protein-induced enterocolitis syndrome. While immunoglobulin E is not involved, the mechanism(s) that result in the food-induced gastrointestinal symptoms are unclear, although T cell activation has been considered. We report four cases of food protein-induced enterocolitis syndrome caused by different solid foods and without concomitant immunoglobulin E sensitization to milk and soya. Clinical and laboratory evidence of type I immunoglobulin E mediated food reactivity and food-induced T cell activation was absent in each case.

**Case presentations:**

Case 1 concerned a 20-month-old South Asian boy who had experienced four episodes of severe vomiting with flaccidity since four months of age and two hours after consuming rice.

Case 2 involved a nine-month-old Caucasian boy who had suffered three episodes of severe vomiting with flaccidity since six months of age and three hours after consuming wheat.

The child in Case 3 was a 16-month-old Caucasian boy who had suffered three episodes of severe vomiting with flaccidity since nine months of age and two hours after consuming cod.

Case 4 involved a 15-month-old South Asian boy who had suffered three episodes of severe vomiting since eight months of age and two hours after consuming chicken.

**Conclusion:**

In children with recurrent marked delayed vomiting after the ingestion of specific foods and in whom bronchospasm, skin rash and angioedema are absent, food protein-induced enterocolitis syndrome should be considered. Skin prick testing and specific immunoglobulin E antibodies are negative and the mechanism of the vomiting is unclear. We speculate whether food protein-induced oligoclonal T cell activation may be present. This has similarities to various animal models and improvement may involve deletion of these T cells.

## Introduction

Food protein-induced enterocolitis syndrome (FPIES) is a relatively rare non-immunoglobulin-E (IgE)-mediated [1] disorder that is frequently misdiagnosed at first presentation [2]. It is characterized by food-induced severe vomiting in all cases, lethargy in over 85%, pallor in two thirds and diarrhea in about a quarter of patients [2]. These bouts typically occur between two and three hours after ingestion of the offending food. In the majority of cases, the attacks start at around six months of age. The most commonly implicated foods include cow’s milk, soya, rice, fish, wheat and chicken. More recently, fruit proteins have also been implicated [3]. In rare cases, even the very small amounts of maternally ingested food proteins that are present in breast milk have induced the characteristic symptoms [4]. We report four cases of FPIES occurring in response to wheat, rice, chicken and cod and show that the symptoms are similar in each of these types of FPIES. Additionally, efforts to establish food-induced T cell activation were negative, suggesting alternative mechanisms of intense gastrointestinal irritation and hypersecretion.

## Case presentations

### Case 1

Between the ages of 4 and 16 months, a 20-month-old nonatopic Pakistani boy had experienced four separate bouts of profuse vomiting approximately two hours after he had consumed rice. The quantity of rice inducing these reactions varied between 10 g with the first reaction to a few grains of rice with the most recent reaction. None of these reactions had been associated with diarrhea, skin rash or angioedema. With three of the reactions, the child had become limp and with one he had briefly stopped breathing. Neither stridor nor wheezing had been evident with the latter reaction. Investigations confirmed a total IgE of 130kU/L (ImmunoCAP; Thermo-Fisher) with negative specific IgE to rice, wheat, oats, soya, cod, egg, potatoes, peas, cauliflower, apple or a nut mix, all giving values <0.1kU_A_/L (ImmunoCAP; Thermo-Fisher, Horsham, UK). Skin prick testing was negative to rice, milk, egg and banana. A graded rice challenge is planned for when the child is four years of age.

### Case 2

Between the ages of six and nine months, a nonatopic Caucasian boy had experienced four bouts of profuse vomiting approximately three hours after the consumption of bread and foods containing wheat. The quantity of wheat inducing these reactions varied between 15 g with the first reaction to a little less than half a gram with the most recent reaction. None of these reactions had been associated with diarrhea, skin rash or angioedema. With one of the reactions, the child had become very limp but none had been accompanied by dyspnea. Investigations confirmed a total IgE of <2kU/L with negative skin prick testing to wheat, milk, soya, corn and cod. A wheat challenge at 21 months of age produced only drowsiness but no vomiting after the consumption of 5 g of bread.

### Case 3

From the age of nine months, a 16-month-old Caucasian boy with mild eczema and asthma had experienced three bouts of profuse vomiting approximately two hours after the consumption of cod. The quantity of cod inducing these reactions varied between 5 g with the first reaction to a little less than a quarter of a gram with the most recent reaction. None of these reactions had been associated with diarrhea, skin rash or angioedema. With one of the reactions, the child had become limp but none had been accompanied by dyspnea. Investigations confirmed a total IgE of 371kU/L with mildly positive specific IgE to cod at 0.98kU_A_/L and haddock at 0.83kU_A_/L (ImmunoCAP; Thermo-Fisher). The result was minimally positive to shrimp at 0.27kU_A_/L and significantly positive to wheat at 36kU_A_/L, oats at 18kU_A_/L and corn at 15kU_A_/L. The child had tolerated these cereals without reactivity on numerous previous exposures. Skin prick testing to cod, salmon, shrimp was, however, completely negative. A graded cod challenge is planned for when the child is four years of age.

### Case 4

From the age of eight months until presentation at 15 months, a nonatopic Pakistani boy had experienced three separate bouts of profuse vomiting approximately two hours after he had consumed well-cooked chicken. The quantity of chicken inducing these reactions varied between 5 g with the first reaction to less than a quarter of a gram with the most recent reaction. None of these reactions had been associated with diarrhea, skin rash, angioedema, dyspnea or flaccidity. Investigations confirmed a total IgE of 86kU/L with negative specific IgE of <0.1kU_A_/L to chicken and egg as well as to beef, mutton, pork, cod and shrimp (ImmunoCAP; Thermo-Fisher). Skin prick testing to chicken and egg was also negative. A graded chicken challenge is planned for when the child is between three and four years of age.

## Methodology for assessing food-induced T cell activation

Peripheral blood mononuclear cells, obtained from venous blood by density centrifugation on Ficoll, were incubated at 37°C and 5% carbon dioxide with several concentrations of the test and control foods. T cell activation was assessed by the expression of CD69 phycoerythrin and CD25 phycoerythrin by CD3 fluorescein isothiocyanate-positive cells analyzed by flow cytometry after fixing in Cellfix. All reagents were obtained from Becton Dickinson Biosciences, San Jose, CA, USA.

## Results

The percentage of activated T cells was assessed using two sets of dual immunofluorescence; CD3 + CD25+ and CD3 + CD69+. In each child both methods of assessing T cell activation showed identical results when the blood samples were incubated with the food inducing the FPIES (3% to 5%) or a food that was tolerated (3% to 5%).

## Discussion

Cow’s milk protein (CMP)- and soya-induced FPIES are the most common FPIES overall and both frequently coexist [5]. CMP FPIES has been reported to have a prevalence rate of 0.34% calculated on the basis of 44 out of 13,019 Israeli infants assessed [6]. Most children outgrow their FPIES by two to three years of age. This was evident in the Israeli study, where 90% of the 44 children had outgrown FPIES by three years of age [6]. Nowak-Wegrzyn *et al.*[7] observed FPIES due to solid foods to resolve at a median age of 24 months (range 14 to 44 months) compared with 28 months (range 14 months to 21 years) for CMP FPIES. The exact timing of oral food challenge (OFC) for CMP has been suggested to be at over 12 months of age and for soya at over 6 to 8 months age [8]. Similar information for FPIES due to solid foods is not presently available. However, we suggest that an OFC should be delayed longest where the quantity of food required to induce the vomiting is smallest, as the number of sensitized immune cells is likely to be greatest in these individuals. For our patients, the timing of the OFC is based on previous reports on the median and range of time when FPIES to solid food resolves [7,9].

Of the solid foods, rice is the most common cause of FPIES [9]. Whether this reflects the higher frequency of rice exposure in early infancy relative to other foods is unclear and it is noteworthy that it is a rare cause of IgE-mediated reactivity. Interestingly, rice-induced FPIES was more frequently associated with fluid resuscitation and a longer delay in diagnosis than FPIES associated with CMP and soya [10]. Apart from this, there do not appear to be any other clinical differences between solid food FPIES and that associated with milk and soya. Other foods reported to cause FPIES include oats, barley, sweet potato, squash and poultry [7]. Legumes other than soya, such as string beans, lentil and peas, have also been reported to cause FPIES [7]. In those with solid food-induced FPIES, nearly four fifths had a sensitivity to one or more other food. This was frequently milk and/or soya, and in the case of those with cereal-induced FPIES, sensitivity to another non-index cereal was evident in half [7]. There is some evidence that FPIES can transform into a typical IgE-mediated food allergy in a small proportion of children [6]. Thus far, this has not occurred in our patient in Case 3, who has demonstrated sensitization and IgE antibodies to cod and haddock.

While the diagnostic criteria for all types of FPIES have not been defined, those for CMP induced FPIES include age less than 9 months at first episode, delayed recurrent vomiting and lethargy after exposure to CMP. As with all types of FPIES, there should be no evidence of those signs typically associated with type I IgE-mediated allergy. These include urticaria, asthma and angioedema. In Case 3, we believe that FPIES was the cause of the profuse vomiting in our patient rather than an IgE-mediated fish sensitivity. This would explain why our patient was negative to cod and salmon on skin testing and he had not suffered any of the symptoms of an IgE-mediated reaction. Interestingly this patient was more highly sensitised to wheat and oats both of which were tolerated without immediate reactivity.

In all cases of FPIES, the attacks of vomiting can be of such ferocity as to lead to hypovolemic shock and hypothermia. At present, there are no confirmatory blood tests. Diagnosis is based on the characteristic history and an in-hospital food challenge performed with full resuscitation facilities at hand, particularly for rapid intravascular volume repletion. A positive OFC in those with FPIES is often accompanied by an increase in the peripheral blood neutrophil and platelet count [5].

At present, there are no *in vitro* tests capable of confirming FPIES and the precise immune mechanisms involved are unclear. The value of atopy patch testing (APT) was investigated by Fogg *et al.*[11] in 19 cases of FPIES. They found APT predicted the results of OFC in 28 out of 33 instances. The APT was positive in 16 cases of a subsequently positive OFC. However, the APT was also positive in five patients where the OFC was negative. Importantly, the APT was negative in all 12 cases where the OFC was also subsequently negative to the suspected food.

In the four children whose cases we have reported, we have been unable to document T cell activation after prolonged stimulation with the relevant food. Three explanations are evident. First, we focused on delayed and not early T cell activation. Second, only a small fraction of total T cells may be activated in FPIES. Third, the food responsive T cells are confined to the mucosa of the upper gastrointestinal tract and are absent in the peripheral blood. However, Mori *et al.*[12] have recently found increased interleukin-4 and reduced interferon-γ after a positive OFC with rice. Six months later with a negative rice challenge there was increased interleukin-10 expression. Previously, van Sickle *et al.*[13] found increased lymphocyte proliferation in children with soy- and milk-induced FPIES returning a positive food challenge compared with those with a negative response. This was not observed by Shek et al. [14] who found lymphocyte proliferation responses to be similar between 113 IgE-mediated CMP allergy patients and 13 that had CMP-induced FPIES. Comparison of the responses between the incriminated food and one that is tolerated in the same infant, as we did, was not undertaken by either of these groups. Interestingly, Shek *et al.*[14] noted significantly reduced IgG4 antibodies to CMP and a trend to increased IgA in those with FPIES when compared with healthy controls [14]. In this regard, it is interesting that Chung *et al.*[15] had earlier found reduced expression of the type 1 but not type 2 transforming growth-β receptor in the duodenal biopsies of 28 patients with OFC-confirmed FPIES who had villous atrophy compared with those who did not. This was also the case with increased tumor necrosis factor-α expression in those with villous atrophy. Collectively, their results suggest impaired immune regulation with increased inflammatory cytokine expression.

In FPIES, the severe vomiting and diarrhea that occurs around two hours after food ingestion suggests very rapid activation of fluid secretion into the upper gastrointestinal tract. Thus, intact or partially digested food proteins are likely involved. There is also some form of direct relationship between the severity of the symptoms and the quantity of food ingested. The villous atrophy that has been recorded in many children with FPIES suggests loss of enterocytes and impaired barrier function as well as tolerance mechanisms. The rapid rise in the neutrophil and platelet count, while suggesting a significant proinflammatory cytokine release, perhaps including interleukin-8, may also be caused by hemoconcentration. Collectively, these changes are similar to models of bacterial superantigen-mediated oligoclonal T cell activation leading to vomiting and diarrhea developed in piglets and ferrets [16,17]. Arachidonic acid products and particularly leukotriene release [18] would appear to be important subsequent mediators leading to fluid secretion within the upper and then lower gastrointestinal tract. Interestingly, amelioration of the vomiting may be expected in FPIES with leukotriene receptor antagonism [18] and ultimate improvement would require deletion of the respective V beta-restricted oligoclonal T cells. The absence of significant T cell activation in FPIES after prolonged food-mediated lymphocyte stimulation suggests that we and previous researchers have focused on the wrong area of lymphocyte activation kinetics. There is no data on very early lymphocyte activation events. Understanding the mechanism is clearly important in determining the best means of treatment and encouraging improvement and tolerance. On present understanding, oral desensitization should not work, although this has not been tried.

## Conclusion

Severe recurrent vomiting occurring two or more hours after feeding and without associated skin rash, angioedema and bronchospasm should raise the possibility of FPIES. Foods such as rice, chicken and wheat that are normally considered rare inducers of allergy are not infrequently involved. Blood and skin tests for IgE-mediated food sensitivity are negative and aggressive fluid resuscitation is sometimes required for hypotension and dehydration. The immune mechanism underlying FPIES is unclear but rapid T cell activation is likely involved.

## Consent

Written informed consent was obtained from the patients’ parents for publication of this case series. Copies of the written consent are available for review by the Editor-in-Chief of this journal.

## Abbreviations

APT, atopy patch testing; CMP, cow’s milk protein; FPIES, food protein-induced enterocolitis syndrome; Ig, immunoglobulin; OFC, oral food challenge.

## Competing interests

The authors declare that they have no competing interests.

## Authors’ contributions

ASB saw the patients, managed them clinically and drafted the report. SB performed the lymphocyte activation and RAB did the literature search and helped write the report. All authors have read and approved the final manuscript.
